# The effect of intravenous iron supplementation on exercise capacity in iron-deficient but not anaemic patients with chronic kidney disease: study design and baseline data for a multicentre prospective double-blind randomised controlled trial

**DOI:** 10.1186/s12882-022-02896-3

**Published:** 2022-07-27

**Authors:** Sharlene A. Greenwood, Nicholas Beckley-Hoelscher, Elham Asgari, Salma Ayis, Luke A. Baker, Debasish Banerjee, Sunil Bhandari, Kate Bramham, Joseph Chilcot, James Burton, Philip A. Kalra, Courtney J. Lightfoot, Kieran McCafferty, Thomas H. Mercer, Darlington O. Okonko, Benjamin Oliveira, Chante Reid, Alice C. Smith, Pauline A. Swift, Anastasios Mangelis, Emma Watson, David C. Wheeler, Thomas J. Wilkinson, Fiona Reid, Iain C. Macdougall

**Affiliations:** 1 King’s College Hospital NHS Trust, London, UK; 2 King’s College London, London, UK; 3 Guy’s and St Thomas’ NHS Trust, London, UK; 4 Department of Health Sciences, University of Leicester, Leicester, UK; 5 St George’s Hospital NHS Trust, London, UK; 6 Hull University Teaching Hospitals NHS Trust, Hull, UK; 7 Royal Hospital, Northern Care Alliance NHS Foundation Trust, Salford, UK; 8 National Institute of Health Research (NIHR) Leicester Biomedical Research Centre (BRC), Leicester, UK; 9 The Royal London Hospital NHS Trust, London, UK; 10 Queen Margaret University, Edinburgh, UK; 11 Epsom and St Helier University Hospitals NHS Trust, London, UK; 12 University College London, London, UK; 13 National Institute of Health Research (NIHR) Applied Research Collaboration (ARC) East Midlands, Leicester, UK

**Keywords:** Iron, Chronic kidney disease, Exercise, Physical activity, Muscle metabolism, Biopsy, Magnetic resonance imaging, Quality of life

## Abstract

**Background:**

Many people living with chronic kidney disease (CKD) are iron deficient, even though they may not be anaemic. The Iron and Muscle study aims to evaluate whether iron supplementation reduces symptoms of fatigue, improves muscle metabolism, and leads to enhanced exercise capacity and physical function. We report here the trial design and baseline characteristics.

**Methods:**

This is a prospective, double-blind multicentre randomised controlled trial (RCT) including 75 non-dialysis stage 3–4 CKD patients with iron deficiency but without anaemia. Patients were randomly (1:1) assigned to either: i) intravenous iron therapy, or ii) placebo, with concurrent recruitment of eight CKD non-iron deficient participants and six healthy volunteers. The primary outcome of the study is the six-minute walk test (6MWT) distance between baseline and four-weeks. An additional exercise training programme for patients in both groups was initiated and completed between 4 and 12 weeks, to determine the effect of iron repletion compared to placebo treatment in the context of patients undertaking an exercise programme. Additional secondary outcomes include fatigue, physical function, muscle strength, muscle metabolism, quality of life, resting blood pressure, clinical chemistry, safety and harms associated with the iron therapy intervention and the exercise training intervention, and hospitalisations. All outcomes were conducted at baseline, 4, and 12 weeks, with a nested qualitative study, to investigate the experience of living with iron deficiency and intervention acceptability. The cohort have been recruited and baseline assessments undertaken.

**Results:**

Seventy-five individuals were recruited. 44% of the randomised cohort were male, the mean (SD) age was 58 (14) years, and 56% were White. Body mass index was 31 (7) kg/m^2^; serum ferritin was 59 (45) μg/L, transferrin saturation was 22 (10) %, and haemoglobin was 125 (12) g/L at randomisation for the whole group. Estimated glomerular filtration rate was 35 (12) mL/min/1.73 m^2^ and the baseline 6MWT distance was 429 (174) m.

**Conclusion:**

The results from this study will address a substantial knowledge gap in the effects of intravenous iron therapy, and offer potential clinical treatment options, to improve exercise capacity, physical function, fatigue, and muscle metabolism, for non-dialysis patients with CKD who are iron-deficient but not anaemic. It will also offer insight into the potential novel effects of an 8-week exercise training programme.

**Trial registration:**

EudraCT: 2018–000,144-25 Registered 28/01/2019.

## Background

In normal healthy individuals, iron deficiency (ID) is defined as transferrin saturation (TSAT) < 16% and serum ferritin < 30 µg/L [1], but in people with chronic kidney disease (CKD), defined thresholds for iron deficiency are higher (ferritin < 100 µg/L or TSAT < 20%), albeit this not evidence-based. Iron is central to oxygen uptake, transport, storage and metabolism in both skeletal and cardiac muscle [2, 3]. Recently there has been increased interest in iron metabolism in skeletal muscle [4], which accounts for 10–15% of total iron body content, where iron is fundamental for oxygen storage in myoglobin, oxidative metabolism, and energy production by iron-containing mitochondrial enzymes. Early work in both animals and healthy individuals [5, 6] has shown that ID results in reduced mitochondrial enzyme activity leading to lower energy production through oxidative phosphorylation; these abnormalities appear to be restored following iron repletion [5, 7, 8].

Traditionally, the greatest pathophysiological concerns with ID were considered to be through deficient haem synthesis, resulting in low haemoglobin concentrations. However, ID in the absence of anaemia may also have adverse effects, particularly in relation to cardiac and skeletal muscle function. This has been most extensively investigated in patients with heart failure in which those treated with intravenous iron have improved exercise capacity and symptoms including patient-reported outcomes [9–11], as early as 4 weeks and lasting up to 24 weeks after treatment compared to those receiving placebo. These findings were also demonstrable up to 12 months follow-up [12] with a significant reduction in hospitalisations. These effects were evident without any change in haemoglobin and assumed to be mediated via improvements in cardiac and/or skeletal muscle function following iron repletion.

However, a recently published study in non-dialysis kidney patients who were non-anaemic but iron deficient did not report any statistical significant beneficial effect of iron therapy on exercise capacity at 4 or 12 weeks, but did show numerical improvements in exercise capacity measurement in the iron therapy group [13]. This trial had a small sample size and there were imbalances in baseline exercise capacity between groups, which may account for the non-significant outcome. To date, there are limited data about the effects of ID on cardiac or skeletal muscle metabolism in patients with CKD [14], but it is proposed that ID contributes to mitochondrial dysfunction and reduced energy production in cardiac and/or skeletal muscle of CKD patients, and importantly may contribute to the reduced exercise capacity, physical function and fatigue commonly reported in this population [15, 16]. Some studies have identified skeletal muscle mitochondrial dysfunction in CKD, which contributes to skeletal muscle dysfunction [17–19]. In addition, exercise training for patients with CKD has been shown to confer a wealth of health-related benefits; however the attenuated training responses sometimes observed to exercise training are often unexplained [20]. Combining exercise training with iron therapy may target the disease-related derangements in the oxygen transport chain and result in more pronounced physiological adaptations to exercise in patients with CKD [20].

Given the importance of fatigue and its consequences on patient outcomes and quality of life, timely and effective management of fatigue represents a clinical priority [16, 21]. We will examine whether a routine strategy of IV iron therapy in patients with stages 3–4 CKD who are iron-deficient (Ferritin < 100 µg/L and/or TSAT < 20%) but *not* anaemic (haemoglobin 110-150 g/L) leads to improvements in exercise capacity. The results of this trial will provide data to ascertain whether intravenous iron therapy is beneficial to exercise capacity, muscle metabolism, physical function, and fatigue in patients with CKD and ID, but without anaemia, and determine its effects after an exercise intervention. We hypothesise that repleting iron stores via administration of intravenous iron will improve skeletal muscle metabolism and reduce fatigue in iron deficient patients with CKD.

## Objectives

### Primary objective

To assess the effect of intravenous iron compared to placebo on exercise capacity, as assessed by the distance walked during the 6-min walk test (6MWT) at 4 weeks post iron infusion.

### Secondary objectives

The secondary objectives were: i) to assess the effect of intravenous iron compared to placebo on physical capacity, quality-of-life, and skeletal muscle metabolism at 4 weeks; and ii) to assess the effect of intravenous iron compared to placebo on physical capacity, quality-of-life, and skeletal muscle metabolism at 12 weeks following an 8-week exercise programme offered to all trial patients.

Specifically, we propose to examine whether a routine strategy of IV iron therapy in patients with stages 3–4 CKD who are iron-deficient leads to improvements in exercise capacity and the following outcomes.

### Biochemical measures

- Iron status (Ferritin, TSAT) and haemoglobin
- Kidney function (urea, creatinine, estimated glomerular filtration rate)

### Physical measures

- VO_2_ peak test (in a sub-set of participants)
- Isokinetic dynamometry (muscle strength of knee extensors)
- Functional capacity (sit-to-stand 60 to assess lower limb function)

### Psychosocial measures

- Quality of life (KDQOL-36)
- Qualitative exploration of participant experience
- The Work and Social Adjustment Scale (WSAS)
- Chalder Fatigue Questionnaire – self-reported physical and mental fatigue

### Trial measures

- Skeletal muscle phosphocreatine recovery half-time (PCr t_1/2_) on MRI spectroscopy
- Muscle metabolism
- Adverse events

### Exploratory objective

An exploratory objective will be to explore the impact or iron therapy on iron regulatory genes (haemochromatosis gene (HFE), Transmembrane proteinase serine 6 (TMPRSS6) and explore any correlations with outcome measures.

## Methods

### Design, setting and participants

This is an investigator-led multicentre double-blind randomised placebo-controlled trial of patients with stages 3–4 CKD and ID, but without anaemia, aged 18 or over.

Brent Ethics Committee approved the protocol (19/LO/0128) and the study was prospectively registered (EudraCT: 2018–000,144-25 on 28/01/2019).

**Fig. 1:**
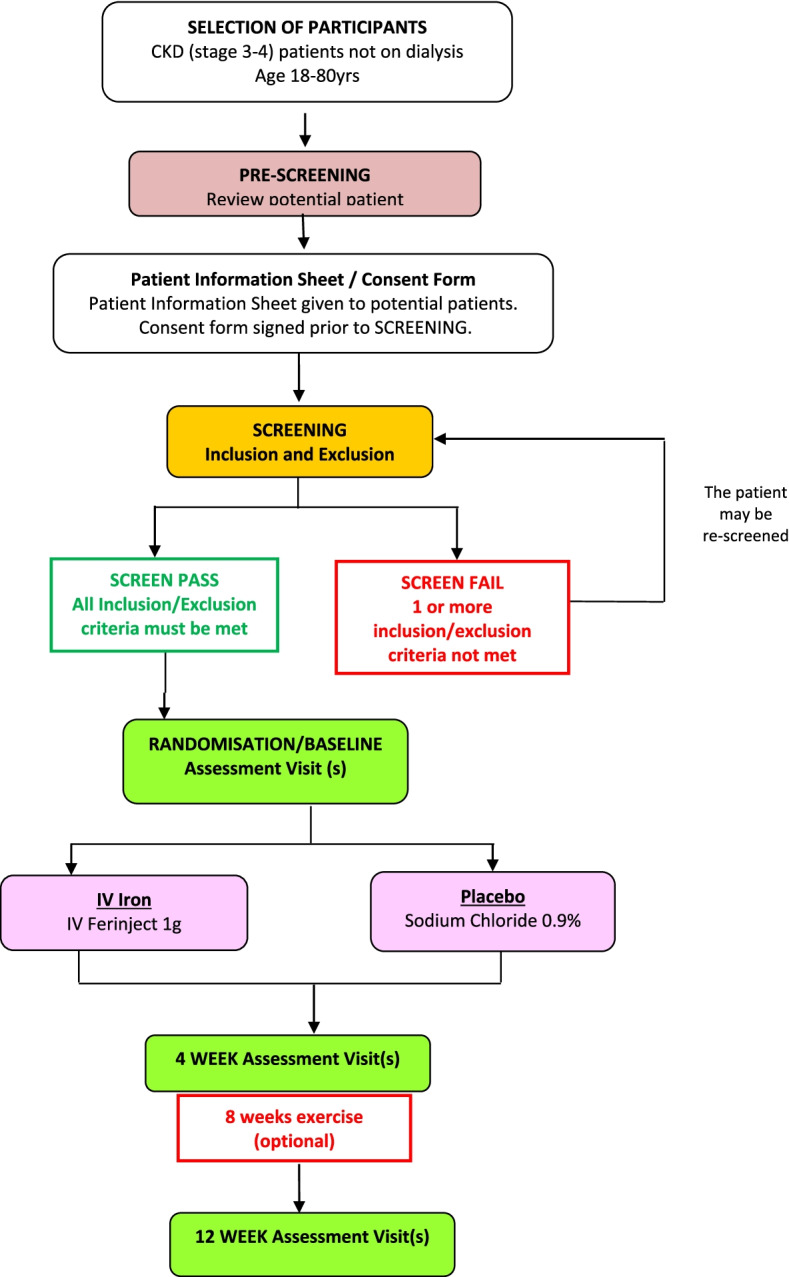
Trial Flowchart

**Fig. 2:**
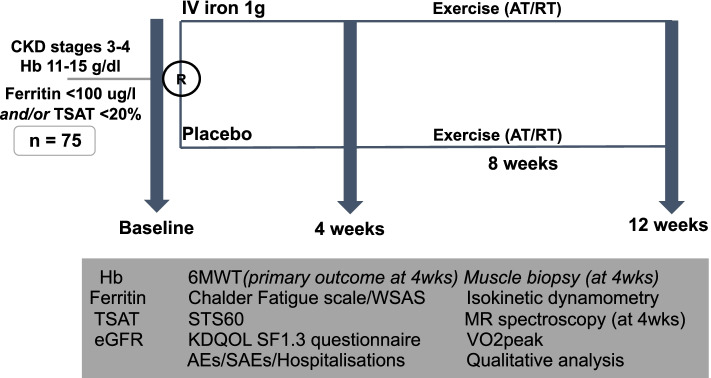
The Iron and Muscle Study design

The flow of participants through the trial including recruitment, randomisation and baseline assessment is summarised in Fig. 1. The study design is summarised in Fig. 2. Participants were recruited from seven renal units in the UK.

### Recruitment and eligibility

Potential participants were identified during routine hospital consultations and concurrent evaluation of clinical records to confirm eligibility for participation. If considered eligible for the study, they were approached by a member of the renal healthcare team who discussed the study and provided a Participant Information Sheet (PIS). After allowing the patient a minimum of 24 h to read and consider the information in the PIS, and to consult with family members, the research team approached the patient to answer any questions. If the participant was agreeable to proceed, an appointment was made for the baseline outcome assessment. Written informed consent was obtained by a member of the research team prior to any study assessments. See Table 1 for inclusion and exclusion criteria.

**Table 1:** Inclusion and exclusion criteria

*Inclusion criteria:*	*Exclusion criteria:*
• Patients with stage 3–4 CKD	• Pregnancy or breast feeding (female patients of childbearing age were asked if there was any possibility, they may be pregnant)
• Resting BP ≤ 160/95 mmHg	• Body mass < 50 kg
• Serum ferritin level less than 100 µg/L AND/OR transferrin saturation ≤ 20%	• Kidney, kidney-pancreas and liver transplant patients
• Haemoglobin 110–150 g/L	• Patients with known allergy to iron therapy
• male or female	• Haemochromatosis, porphyria cutanea tarda or history of acquired iron overload
• Aged 18–80 years	• History of intravenous iron within previous 6 weeks
• Able to provide written informed consent	• Patients with CRP > 50 mg/L
	• Patients with serum phosphate < 0.7 mmol/L
	• Active infection
	• Current therapy with ESAs
	• Current oral, intravenous or topical (on legs) immunosuppressive treatment or use within previous 3 months
	• Uncontrolled atrial fibrillation
	• Use of anticoagulants in those under consideration for muscle biopsy
	• Unstable angina or heart attack within the last year
	• Presence of solid organ cancer
	• Known haemoglobinopathy, myelodysplasia, or myeloma
	• Patients with peripheral vascular or musculoskeletal disease who the investigator deems unable to carry out the 6MWT
	• Patients with known severe aortic stenosis or pacemaker in-situ
	• History of severe atopy
	• Severe liver disease with serum transaminases > × 3 upper limit of normal range according to local laboratory values
	• Severe lung disease with FEV_1_ known to be < 50% predicted in last year
	• Known heart failure with a left ventricular ejection fraction < 45% in last year
	• Any other health condition considered by the local Principal Investigator to be a contraindication to IV iron

### Iron randomisation and blinding

Patients were randomised within 4 weeks of passing the screening requirements and were assigned in a 1:1 ratio using a secure web-based service through King’s Clinical Trials Unit. Randomisation used an approach based on randomly varying block sizes. Randomisation was stratified by a single binary variable, defined by whether patients had a screening ferritin > 50 µg/L or ≤ 50 µg/L. The number of patients who went forward to randomisation was capped so that there was a maximum of 35 patients had a screening ferritin > 50 µg/L.

The Ferric Carboxymaltose (Ferinject®) solution is dark brown in appearance; blinding was achieved by shielding the patients from seeing preparation of the study drug and having unblinded study personnel not involved in any study outcome assessments responsible for preparing and administering the study treatment. The study drug was prepared and administered behind a screen or curtain, and the drug administered through normal IV tubes and shielded from vision by use of light protection giving set covers. Trial-specific blood tests taken after IV iron or placebo administration was withheld from the hospital’s electronic patient records to maintain investigator blinding, in particular from ferritin levels. These blood test results were also held in a secondary secure trial database.

### Interventions

Participants were randomised to receive either ferric carboxymaltose (Ferinject®) or placebo at the baseline visit. All participants were offered eight weeks of exercise training between week 4 and week 12 assessment visits. Participants allocated to the placebo group were followed up in routine clinic after completing the trial and offered standard treatment, including treatment for their iron deficiency if still present.

#### Ferric carboxymaltose (Ferinject®) group

Participants in this group received a total iron dose of 1000 mg Ferinject® as a one-off infusion in 100 ml normal saline administered over a minimum of 15 min.

#### Placebo group

Participants in this group received 100 ml normal saline administered over a minimum of 15 min.

#### Exercise training

Participants were assessed at 4-weeks and offered three 1-h sessions of exercise training each week for 8 consecutive weeks, concentrating on large muscle groups of the lower limbs. Following a 6-min warm-up on the cycle ergometer (3 min of warm up at 1/3 of training workload at constant and rhythmical pace and 3 min of warm up at 1/2 of training workload), training involved a combination of cycle ergometry and resistance training, the latter performed on the isokinetic dynamometer machine Biodex, Mirion Technologies (Biodex), Inc. USA. To conclude, patients spent 6 min cooling down on the cycle ergometer (3 min of cool down at 1/3 of training workload and 3 min of cool down with very little or no resistance at slower but constant pace). Both the intensity and the duration of the exercise programme were progressively increased, as individually tolerated. Cycle ergometry was progressed from 65 to 80% of peak watts achieved during testing, determined during a graded incremental exercise test performed to peak endurance capacity, and monitored with heart rate monitors. Resistance training involved patients performing 3 sets of 10 repetitions with a 30 s rest between sets. Isokinetic knee extension at 90 degrees/sec was completed at 65–85% of the maximum torque achieved at 90 degrees flexion, which was determined using the isokinetic dynamometer. Each participant’s training programme was re-assessed and progressed at 4 weeks into the programme. The intervention was delivered by trained physiotherapy assistants, on an individual basis, in a hospital gym.

#### Adjusted exercise training due to COVID-19

Due to Covid-19 related restrictions on study visits, and related patient concerns about travelling and attending hospital, patients were offered the option of completing these sessions of exercise training at home via an online kidney-specific exercise platform, called Kidney Beam.

https://beamfeelgood.com/onDemand/list/kidney-disease that was available on any technological device. All participants were offered support to sign-up to the platform and engage with the resource. Those participants who did not have access to any technological device, were offered a loan of a tablet. Each participant was provided with a heart rate monitor for use during the 8-week exercise training programme, written guidance on how to access the platform, and exercise recording sheets.

### Assessment outcomes and their measurement

#### Baseline clinical information and study visits

Following screening, willing, and eligible participants were invited to attend a baseline visit, at which baseline investigations were performed (Table 2**)**. The baseline/randomisation visit(s) occurred no more than four weeks after the patient had successfully passed a screening visit. The assessments were split over two visits if the participant preferred. Demographic data (date of birth, sex, ethnicity, smoking status, alcohol intake, body mass index (BMI), and primary aetiology of CKD were collected and recorded. A detailed disease history including cardiovascular co-morbidity and list of concomitant medications was taken. Vital signs were recorded including blood pressure. A physical examination was not required.

**Table 2:** Overview of trial schedule

**Procedure**	**Screen Visit**	**Baseline** **Visit 1** **(Local site)**	**Baseline Visit 2** **(KCH)**	**Week 4** Visit 1 (Local)	**Week 4 Visit 2 (KCH)**	**Week 12** **Visit 1 (Local)**
		Within 4 weeks of Screening visit		4 weeks ± 4 days from IMP Administration		12 weeks ± 14 days from IMP Administration
Patient information and informed consent	X					
Inclusion / Exclusion criteria	X					
Demographic data and medical history	X					
Concomitant medications	X	X	X	X	X	X
Vital Signs	X	X	X	X	X	X
Weight	X					
Height, weight, BMI, waist-to-hip ratio		X		X		X
IMP administration			X			
Exercise prescription				X		
Full blood count	X			X		X
Ferritin & TSAT	X			X		X
U + E, Creatinine, LFTs, CRP	X			X		X
eGFR, Phosphate	X			X		X
Soluble Transferrin Receptor and stored sera/plasma/whole blood (EDTA)			X		X	X
Exercise capacity (6MWD, VO_2_ peak)		X		X		X
Muscle strength (isokinetic dynamometry)		X		X		X
Fatigue Severity Score (Chalder Fatigue Questionnaire)		X		X		X
Functional impairment (WSAS Questionnaire)		X		X		X
Quality of life (KDQOL-36)		X		X		X
Functional capacity (STS60)		X		X		X
MRI spectroscopy (subset of participants)			X		X	
**Procedure**	**Screen Visit**	**Baseline** **Visit 1** **(Local site)**	**Baseline Visit 2 (KCH)**	**Week 4** **Visit 1 (Local)**	**Week 4 Visit 2 (KCH)**	**Week 12** **Visit 1 (Local)**
		Within 4 weeks of Screening visit		4 weeks ± 4 days of IMP Administration		12 weeks ± 14 days of Baseline visit
Muscle metabolism via muscle biopsy (subset of participants)			X		X	
Qualitative Interview		X			X (if no exercise)	X
Adverse Events		X		X		X
Whole blood DNA (EDTA) *(optional)*			X			

Baseline investigations included estimated glomerular filtration rate (eGFR) and biochemical profile (BCP), full blood count (FBC), serum ferritin (SF), transferrin saturation (TSAT) and C-reactive protein (CRP). Quantification of proteinuria was carried out by measurement of urinary protein:creatinine ratio (uPCR) or if diabetic, urinary albumin:creatinine ratio (uACR) levels in a spot urine sample using standard laboratory techniques.

Muscle biopsies for participants and controls were taken on a separate visit (baseline visit 2) as they needed to be collected following 1–2 days of physical inactivity. Patients from the other London sites participating in the muscle biopsy/MRI sub-study attended for the baseline visit 2 assessments at King’s College Hospital.

Qualitative interviews at entry to the trial were preferably completed on a further separate visit but could be combined with the main baseline or muscle biopsy visit if the participant preferred and was completed prior to administration of iron therapy or placebo. Interviews were conducted either face-to-face, in a quiet room separate from where the visit commenced, or via telephone depending upon the preference of the participant.

### Follow-up visits

Patients completed follow-up measures at 4 weeks (± 4 days) and 12 weeks (± 14 days) after treatment.

### Six minute walk test

The primary endpoint for this study was the change in 6MWT distance between baseline and 4 weeks. The outcome was also performed at 12 weeks following the 8-week exercise training programme. Distance (in metres) was measured during a self-paced, standardised 6MWT conducted around a level, 30.5 m circuit [22]. The walk test was completed twice following sufficient rest (20 min) to allow heart rate to return to resting. The results from the second test were used for analysis.

### Muscle strength testing procedure

Knee extensor isokinetic and isometric strength was measured bilaterally using a Biodex S4 Isokinetic System Pro dynamometer Mirion Technologies (Biodex), Inc. USA. Following familiarisation, patients were seated with the lateral epicondyle of the knee aligned with the axis of rotation of the dynamometer and were instructed to perform 3 sets of 5 maximal extension contractions at angular velocities of 60 degrees, 90 degrees and 120 degrees per second. The patient had a 60 s rest between each angular velocity. The highest peak torque achieved in newton meters was recorded. Isometric maximum voluntary contraction was measured using computerised dynamometry with the leg fixed at a 90 degree angle. Patients performed 3 maximal contractions with a 60 s rest between each set, and the highest peak torque in newton meters was recorded.

### Sit to stand 60

Physical function was assessed by the sit-to-stand-60 (STS60) test [23], an accurate and valid measures of lower leg strength and physical function.

### Peak aerobic capacity (in sub-set of patients).

VO_2_ peak (volume of oxygen consumption) was determined by an incremental cycling exercise tolerance protocol. Breath-by-breath gas exchange was measured using cardiopulmonary exercise testing equipment calibrated prior to each patient assessment. The exercise testing protocol started from a 3-min unloaded cycle; followed by ramp increases in resistance of 15 watts/min until one of the following occurred: (i) a plateau in oxygen uptake, (ii) attainment of respiratory exchange ratio ≥ 1.15, or (iii) patient requested to stop. Average oxygen uptake of the final 20 s of the test was recorded as the VO_2_ peak (L/min). Electrocardiogram and heart rate were continuously monitored, and blood pressure (BP) was recorded every one or two minutes throughout the ramp incremental test. Rate of perceived exertion (RPE), using the CR100 RPE scale, and angina scale were recorded every minute for safety.

### Muscle biopsy procedure

Forty participants, eight iron replete controls, and six healthy controls from London-based hospitals were invited to attend King’s College Hospital for the muscle biopsy procedure at baseline and 4 weeks. Patients were asked to refrain from eating or drinking anything other than water on the morning of the biopsy. A muscle biopsy (~ 80-100 mg, the size of a pea) was taken from the Vastus Lateralis muscle of the quadriceps group. With the patient lying supine, the skin of the thigh was thoroughly cleaned using iodine solution, and 4-5 mL 1% lignocaine was injected subcutaneously as local anaesthetic. A small incision (< 0.5 cm) was then made through the skin, subcutaneous fat and muscle fascia at a mid-thigh and lateral position. Through this position a 11-guage microbiopsy needle was inserted and a small sample of muscle taken. Depending upon the amount of tissue collected, a second pass of the needle was performed as required from the insertion point but in an alternative orientation to avoid repeat sampling of a single site. All biopsies were performed by a trained clinician who had prior experience of the muscle biopsy procedure, and all samples were stored and processed as appropriate for subsequent analysis.

Analytical laboratory measurement of factors relevant to muscle metabolism, mitochondrial function and tissue pathology were conducted, including but not limited to:

- Mitochondrial respiration/Oxidative phosphorylation & fuel utilisation measured via OROBOROS.
- Iron regulatory mRNA gene expression measured by qPCR (For example, Hepcidin, Hemojuvelin, Ferroportin, Trf1 receptor, and iron regulatory proteins-1 & -2).
- Protein analysis of mitochondrial abundance and biogenesis related proteins by western blot (For example, OXPHOS complex I to V, PGC1a, Porin, Atp5al, Mdufa9).
- Mitochondrial DNA copy number as an indication of mitochondria mass.
- Protein and mRNA gene expression of molecules related to oxidative stress (for example, SOD1/SOD2, Catalase) and inflammation (for example, TNF-alpha, IL-6).
- Skeletal muscle morphology including fibre typing, cross sectional area & myoglobin density measured by immunocytochemistry.
- Mitochondrial number, appearance & cristae density measured by electron microscopy.

In addition to the CKD trial patients, a control population of 8 CKD non-iron deficient participants and 6 healthy volunteers attended a baseline visit to baseline comparative analysis and to contextualise findings from the intervention trial.

### Circulating factors in serum/plasma

Analytical laboratory measurements including but not limited to factors relevant to iron metabolism (eg. Transferrin), inflammation (eg. IL-6, TNF-a), oxidative stress (eg. TBARs) and metabolic/vascular health will be performed. Samples will be analysed at accredited external laboratories as required under service level agreements between King’s College Hospital and external contracted parties.

### MRI spectroscopy

Forty participants were invited to attend MRI spectroscopy visit at baseline and 4 weeks. Skeletal muscle PCr t1/2 was measured using 31phosphorous magnetic resonance spectroscopy (^31^P-MRS) on a Phillips Achieva MR scanner. This non-invasive technique enables real-time quantification of skeletal muscle energetic molecules (PCr and adenosine triphosphate ATP) during exercise and recovery in-vivo. During exercise, PCr is progressively depleted and then rapidly resynthesized during recovery. Because PCr replenishment is performed exclusively by mitochondria, the rate of PCr recovery after exertion is proportional to mitochondrial function. In addition to the CKD patients, a control population of 8 CKD non-iron deficient subjects and 6 healthy volunteers attended a baseline visit to further validate this technique. The MRI spectroscopy was performed at the King’s College London (KCL) MRI unit located at St Thomas’ Hospital by KCL staff and the research fellow. Where possible, patients having MRI spectroscopy imaging also had muscle biopsies.

### Patient reported outcomes: fatigue, functional impairment, and quality of life questionnaires

Participants were asked to complete the following questionnaires at baseline to capture data about fatigue, functional impairment, and quality of life:

- The Chalder Fatigue Questionnaire is an 11-item questionnaire measuring the severity of physical and mental fatigue on two separate subscales
- The Work and Social Adjustment Scale is a valid and reliable self-report scale of functional impairment attributable to an identified problem (i.e. fatigue)
- The Kidney Disease Quality of Life short-form 1.3 questionnaire is a preference-based measure of health (KD QoL-SF-36) questionnaire, a standardized survey used to assess patient health [24].

Questionnaires were repeated at 4 and 12 weeks.

### Safety and monitoring of the intervention

Serious adverse events were identified and documented on the eCRF at routine visits, based on participant reports and primary or secondary care reports. In addition, episodes of infection requiring hospitalisation and other infection episodes and cases of vascular access thrombosis were documented. In addition, haemoglobin, ferritin, platelet levels, and other laboratory measurements detailed above were monitored.

### Other outcomes

#### Qualitative Study – people living with chronic kidney disease

Individual semi-structured interviews were conducted to understand the impact of CKD on the lived experience, expectations of the iron and exercise interventions before the trial, and their experiences afterwards. A sample of 20 participants were interviewed at entry to and exit from the study. Participants were selected using purposive sampling to ensure varied experiences and viewpoints are represented, for example, including people in both study arms, and including people with different participation rates and response to the intervention. Interviews were conducted face-to-face or via telephone, according to participant preference, and lasted approximately one hour. Face-to-face interviews were conducted in a private room, on a separate visit or combined with the baseline visit depending on participant preference. The interviews were digitally recorded and transcribed verbatim by a professional transcription service. Thematic analysis (Braun and Clarke, 2006) was used to interpret the data and identify themes. The data was stored and managed in QSR NVivo software.

### Sample size

#### Primary outcome: 6MWT distance

The choice of endpoint measures to be studied were chosen to provide clinical relevance. Studies that have evaluated the clinically meaningful change in the distance walked on the 6MWT in patients with heart failure [25, 26] indicate that a clinically meaningful improvement in 6MWT distance is between 32 and 45 m. Calculations from the FAIR-HF study [27] and a study in patients with CKD [28] suggested a clinically meaningful improvement in 6MWD of up to 40 m. We have therefore taken this to be the mean clinically important difference (MCID) at the 4-week primary outcome endpoint. An estimated sample size to detect a clinically meaningful difference of 40 m with a SD of 56 m (derived from the higher SD reported by Tang et al. [28]) at 80% power and 5% alpha revealed a requirement of 62 participants. We aimed to recruit at least 70 patients to enable dropouts and ensure that 4-week primary outcome data were collected for at least 62 patients.

### Other outcomes

(i) MRI spectroscopy: Using unpublished data from the FERRIC-HF II (Okonko DO) trial assessing the impact of iron repletion on skeletal muscle phosphocreatine recovery in anaemic and non-anaemic heart failure patients, mean (± SD) phosphocreatine recovery halftime at study end was 33 ± 7 s in the non-anaemic cohort. To detect a difference of 7 s at 80% power and 5% alpha, we needed at least 32 patients in total. We planned to recruit 40 patients from the total sample to undergo MRI spectroscopy analyses to enable drop-out and insufficient imaging quality.

ii) Muscle biopsy: We also estimated that 40 patients from the total sample would be needed to undergo a muscle biopsy procedure at baseline and four weeks, to enable 16 participants in each arm to detect differences in proposed analyses allowing for drop-out and insufficient sampling. The team has previously performed three exercise intervention studies [29, 30] with sample sizes in each arm ranging from 11 to 18 participants. In all these studies, the team were able to detect significant differences following exercise at the molecular level.

iii) Muscle biopsy phosphocreatine recovery: Using unpublished data from the FERRIC-HF II (Okonko DO) trial assessing the impact of iron repletion on the rate of muscle oxygen consumption in heart failure patients, mean (± SD) ex-vivo muscle oxygen consumption at study end was 0.58 ± 0.22 micromol/min/mg. To detect a meaningful difference of 0.25 micromol/min/mg skeletal muscle phosphocreatine recovery at 80% power and 5% alpha, at least 26 participants are needed, thus we aimed to recruit at least 36 patients to allow for dropouts and patients with poor quality muscle biopsies.

### Statistical analysis plan

#### Descriptive analyses

The results will be constructed according to CONSORT. To assess the comparability of randomised groups at baseline, descriptive summaries of clinical and socio-demographic variables will be presented for each study group. Means and SD will be presented for quantitative variables, and numbers and proportions for categorical variables. No significance tests will be undertaken. If any quantitative variables are highly skewed, medians and interquartile ranges will also be presented.

Descriptive summaries will similarly be provided for each outcome variable, at both 4 weeks and 12 weeks, by study group. The proportions of participants with missing data for each outcome variable will be presented by study group. Baseline characteristics of those with missing follow-up data for key outcomes will be compared descriptively to those with complete data.

Adverse events and serious adverse events (SAE), including serious adverse reactions (SAR) and suspected unexpected serious adverse reactions (SUSAR) will be tabulated. The reasons for any withdrawals from the trial will be summarised.

### Analysis of primary outcome

The difference in means for distance walked during 6MWT (metres) at 4 weeks (primary outcome) between patients randomised to IV iron and placebo will be analysed using an ANCOVA model, using baseline 6MWT distance and the binary stratification variable ferritin (defined as whether or not baseline ferritin is over 50 µg/L) as covariates [31]. Data will be analysed under intention-to-treat assumptions. The mean difference and associated confidence interval will be reported. All analysis will be undertaken in Stata version 15, or a later version if available.

### Analysis of secondary outcomes

The ANCOVA described above for the primary outcome will also be performed for the 6MWT distance (metres) at 12 weeks, and for the other secondary endpoints described earlier. In each case, the baseline value of the variables and the binary stratification factor will be used as covariates.

### Analysis of exploratory outcomes

The impact of each patient’s iron regulatory genotype (e.g., HFE and TMPRSS6) on the primary and secondary objectives of this study will be assessed by adding an interaction term between genotype and trial arm. If any evidence of an interaction is found (e.g., p < 0.1) then we will also report the stratified results.

### Comparison with control groups at baseline

We will explore differences at baseline in muscle metabolism (from muscle biopsy data) and MRI outcomes (from MRI spectroscopy) between the six Healthy Volunteers (HV), eight non-iron deficient CKD controls (CKD) and 40 iron deficient CKD randomised patients using one-way ANOVA. If data are highly skewed, transformation will be considered (e.g. log transformation). If differences are found, post-hoc analysis will be used to determine where differences lie between groups.

### Statistical considerations

#### Time points

Data recorded outside of these time windows will be included in the primary ITT analysis. However, a sensitivity analysis excluding these data points will be performed to assess if this makes any considerable difference to the model results.

#### Stratification

As the stratification variable was necessarily dichotomised in the design stage, the ANCOVA will also use the dichotomised form. However, sensitivity analysis using the continuous form of the stratification variable will also be performed to ensure substantive conclusions are not highly dependent on the dichotomisation [32].

#### Clustering

It is unlikely that there will be large variation between centres on the primary outcome. Therefore, as this is a relatively small trial with a low number of centres, and some centres with a small number of patients, it is unlikely that any small variation between centres can be accurately modelled, and so centre will not be included in the analysis as a fixed effect. However, variation in centre can be included as a random intercept as a sensitivity analysis without sacrificing many additional parameters, an approach that often has advantages over fixed effect models [33].

#### Missing items in scales and subscales

Questionnaire-based variables to be analysed are the Chalder Fatigue Questionnaire, KDQOL-36 [16], and the Work and Social Adjustment Scale (WSAS). The number (%) with complete data will be reported. Where questionnaire-specific guidance is available for the treatment of missing items in the construction of scales, this will be used. Where such guidance is not available, the King’s Clinical Trials Unit guideline will be followed, i.e. scales will be pro-rated for an individual if 20% or fewer items are missing. For example, in a scale with 10 items, pro-rating will be applied to individuals with 1 or 2 items missing. The average value for the 8 or 9 complete items will be calculated for that individual and used to replace the missing values.

#### Missing baseline data

We expect baseline 6MWD data to be nearly complete, if not fully complete. It is unlikely that excluding any patients with missing baseline data will bias treatment effect estimates or lead to much loss of precision, if any [34], and so a complete case analysis will be undertaken in such a scenario.

#### Missing outcome data

If there are missing data for the primary outcome of 6MWT at 4 weeks, several strategies will be considered. If missingness is below 5%, and missingness is not thought to be isolated to a particular clinically relevant subgroup, then a complete-case analysis (CCA) will be performed [35]. If there is more than 5% missing data, then alternative approaches will be used.

If participants with missing 4-week 6MWT data have post-randomisation variables predictive of missingness of 4-week 6MWT (e.g., 12-week 6MWT) *and* predictive of the 4-week 6MWT values themselves, then a linear-mixed model (LMM) will be performed. This will assume that the missing data is missing at random (MAR) conditional on these post-randomisation variables. Unlike CCA, a LMM can include potentially informative post-randomisation variables without impacting the treatment effect estimates. LMMs also perform similarly to multiple imputation (MI) in such a multivariate scenario (SAP15), whilst not requiring completeness of the post-randomisation auxiliary variables. Although it is unlikely that most participants with missing 4-week 6MWT will have 12-week 6MWT data, there may be other post-randomisation variables associated with 4-week 6MWT and its missingness.

If such post-randomisation variables are not present, patterns of missingness in *baseline* variables will be assessed. If the data is thought to be MAR conditional on observed baseline variables, then a complete-case analysis (CCA) adjusted for variables predictive of missingness will be performed. Under the MAR assumption, this gives unbiased and efficient treatment effect estimates, and generally performs better than alternative methods like MI [34].

If there is evidence of an interaction being predictive of both the outcome and missingness of the outcome (e.g., see “Method for handling the impact of COVID-19” section below), then MI will be used instead of the LMM and adjusted-CCA approaches outlined above. MI has the flexibility to handle both the interaction term and the other variables predictive of missingness and gives an unbiased estimate of the average treatment effect (ATE) in the analysis model [34].

The impact of the MAR assumption in all the above approaches will be assessed via a sensitivity analysis, in which a range of plausible values for the missing outcome data will be assumed, and corresponding treatment effect ranges reported.

For the primary outcome variable (6MWT), there may be cases where the participant has recorded a value for the first test but does not have a value for the second test (which is what will be used in the analyses). As it is likely such values will be generated under a missing-not-at-random mechanism, these values will not be included in the primary analysis.

#### Method for handling non-compliance (per protocol/CACE analyses)

The IV iron or placebo administration comprises a single intravenous bolus, given by the unblinded research nurse at the baseline visit (or shortly after) as per the prescription. Non-adherence with treatment is therefore likely to be low, and so no per protocol/CACE analyses will be undertaken.

#### Model assumption checks

Residual plots in all ANCOVA models described above will be checked for normality and homoscedasticity and inspected for outliers and influential points. If any outcome measures have a highly skewed distribution, a transformation of the outcome (e.g., logarithm) may also be considered. The primary outcome (6MWT) is considered likely to be normally distributed.

If there are high degrees of heteroscedasticity then robust standard errors will be considered. If there are any strong outliers then nonparametric (e.g. re-sampling) methods will be considered as an alternative.

#### Method for handling the impact of COVID-19

The characteristics of patients recruited before and after the COVID-19-related study pause will be compared, as suggested by Meyer et al., 2020 [36].

We will first investigate whether the binary variable of being recruited before or after the study break is associated with the primary outcome of 6MWT at 4-weeks. If so, we will see whether this change is additive (e.g., patients post-break walk on average 50 m less than patients pre-break) or multiplicative (e.g. patients post-break walk on average 10% less than patients pre-break). If additive, then it is likely there is no interaction, and so a binary variable indicating their before/after break status will be included as a covariate in the outcome model as a sensitivity analysis. If multiplicative, then this would suggest there may be an interaction. In this case we will perform a sensitivity analysis by adding an interaction term between treatment arm and before/after break status. If there is some evidence of an interaction (e.g., *p* < 0.1) then we will also present the results stratified by before/after break status as a further sensitivity analysis.

The same procedure above will also be performed for the 12-week 6MWT and other outcome variables too. The 12-week 6MWT outcome may be more likely to have an interaction between treatment arm and before/after break status, due to the change in delivery format of the exercise training taking place between weeks 4 and 12. Some of the questionnaire-based outcomes may also be associated with before/after break status.

With regards to missing data, any patients with missing outcome data due to a COVID-19-related site closure will be treated as missing completely at random (MCAR) and excluded from the final primary analysis.

We anticipate that the number of patients becoming infected with COVID-19 during the 4-week window between baseline and the primary outcome timepoint will likely be very low, and so any patients with a missing primary outcome due to COVID-19 infection will be excluded [35]. A similar rationale will also be assumed for all other outcome variables in this case.

If before/after break status is associated with missingness of an outcome and associated with the outcome value itself (but no interaction), then before/after break status will be treated as a baseline variable predictive of missingness as described in the “*Missing outcome data*” section above and included as a covariate in a CCA.

If before/after break status is associated with missingness of an outcome, *and* there exists an interaction between this status and the treatment arm, then MI will be used to handle missing data in the primary analysis, as described in the “Missing outcome data” section above. The imputation model will include this interaction as a covariate, together with other variables predictive of missingness and of the outcome.

### Data monitoring and quality assurance

The trial is coordinated by a Trial Management Group (TMG). A Trial Steering Committee (TSC) was established to oversee conduct and progress of the trial. An Independent Data Monitoring Committee (IDMC) monitored patient safety and treatment efficacy data.

### Baseline characteristics of randomised participants

The baseline characteristics are presented in Tables 3 and 4. 75 participants were randomised including 33 (44%) males. The mean (SD) age was 58 (14) years. 56% of the randomised population was white, 20% was Asian, and 21% was Black African/Black Caribbean. The mean (SD) weight was 84 (18) kg, and most participants were classified as obese with a mean (SD) body mass index 31 (7) kg/m^2^. Serum ferritin was 59 (45) μg/L, transferrin saturation was 22 (10) % and haemoglobin was 125 (12) g/L at randomization for the whole group. Mean (SD) serum creatinine was 175 (71) μmol/L and eGFR was 35 (12) mL/min/1.73 m^2^. The mean (SD) 6MWT distance was 429 (174) m.

**Table 3:** Mean (SD) and Median (IQR) baseline characteristics in all randomised participants (intention to treat population)

Characteristic	N	*N* = 75^a^
Sex	75	
Male		33 (44%)
Female		42 (56%)
Age at registration	75	58 (14), 58 (50, 69)
Ethnicity	75	
White		42 (56%)
Asian		15 (20%)
Black		16 (21%)
Mixed		1 (1.3%)
Other		1 (1.3%)
Smoking status	71	
Current smoker		6 (8.5%)
Ex-smoker		12 (17%)
Non-smoker		53 (75%)
Main cause of renal failure	74	
Diabetic nephropathy		12 (16%)
Glomerular disease		2 (2.7%)
Hypertension		20 (27%)
Tubulointerstitial disease		3 (4.1%)
Renovascular disease		1 (1.4%)
Polycystic kidney disease		11 (15%)
Other		16 (22%)
Unknown cause		9 (12%)
History of kidney transplantation	69	3 (4.3%)
History of myocardial infarction	74	2 (2.7%)
History of ischemic heart disease	74	5 (6.8%)
History of stroke/TIA	74	1 (1.4%)
History of heart failure	74	0 (0%)
History of peripheral vascular disease	74	2 (2.7%)
History of atrial fibrillation	74	0 (0%)
History of hypertension	74	50 (68%)
History of diabetes	74	22 (30%)
History of cancer (excluding skin cancers)	74	4 (5.4%)
History of hyperlipidaemia	74	26 (35%)

^a^n (%); Mean (SD), Median (IQR)

**Table 4:** The mean (SD) and median (IQR) baseline clinical values in all randomised participants (intention to treat population)

Characteristic	N	*N* = 74^a^
Haemoglobin (g/L)	71	125 (12)
Platelet count (× 10^9/L)	71	236 (62), 229 (197, 281)
Ferritin (ug/L)	69	59 (45), 52 (35, 71)
Transferrin saturation (TSAT) (%)	70	22 (10), 20 (17, 25)
Urea (mmol/L)	66	11.6 (5.4), 10.0 (8.0, 14.0)
Sodium (mmol/L)	72	139.79 (2.33), 140.00 (138.00, 141.00)
Potassium (mmol/L)	72	4.57 (0.60), 5.00 (4.00, 5.00)
Creatinine (umol/l)	72	175 (71), 140 (126, 228)
Serum phosphate (mmol/L)	67	1.15 (0.20), 1.10 (1.00, 1.30)
Serum albumin (g/L)	73	42.85 (2.86), 43.00 (41.00, 45.00)
Total Protein (g/L)	54	70.8 (5.4), 71.0 (68.0, 74.0)
CRP (mg/L)	68	4.4 (4.3), 3.0 (2.0, 5.0)
eGFR (ml/min/1.73m^2^)	72	35 (12), 37 (24, 43)
SBP	72	134 (16), 135 (124, 144)
DBP	72	78 (10), 79 (73, 85)
Heart rate (bpm)	72	76 (13), 75 (67, 86)
Height (cm)	72	166 (11), 165 (158, 173)
Weight (kg)	72	84 (18), 80 (72, 92)
Waist circumference (cm)	71	105 (16), 105 (96, 114)
Hip circumference (cm)	71	112 (13), 109 (103, 120)
Body mass index	72	31 (7), 29 (26, 35)
6MWT Distance (metres)	70	429 (174), 480 (360, 543)
STS60 (Number of repetitions)	70	24 (8), 24 (18, 29)
VO2 peak (L/min)	45	1.36 (0**.**48), 1.34 (0.99, 1.62)
VE/VCO2 slope (mL/kg/min)	45	33.5 (5.5), 32.6 (29.2, 36.3)
Muscle strength Right Leg: Highest peak torque achieved at 60 degrees angular velocity (newton meters)	70	97 (44), 90 (68, 117)
Right Leg: Highest peak torque achieved at 90 degrees angular velocity (newton meters)	70	94 (43), 88 (67, 123)
Right Leg: Highest peak torque achieved at 120 degrees angular velocity (newton meters)	70	84 (39), 77 (58, 110)
Right Leg: Isometric maximum voluntary contraction (90-degree angle): Highest peak torque (newton meters)	70	132 (66), 118 (85, 163)
Left Leg: Highest peak torque achieved at 60 degrees angular velocity (newton meters)	67	92 (44), 86 (61, 120)
Left Leg: Highest peak torque achieved at 90 degrees angular velocity (newton meters)	67	83 (39), 79 (51, 107)
Left Leg: Highest peak torque achieved at 120 degrees angular velocity (newton meters)	67	76 (33), 74 (48, 95)
Left Leg: Isometric maximum voluntary contraction (90-degree angle): Highest peak torque (newton meters)	67	124 (61), 114 (79, 150)
Right leg dominant leg	68	55 (81%)
Chalder Fatigue Score	73	17.2 (5.7), 16.0 (12.0, 22.0)
WSAS total score	72	15 (12), 12 (6, 24)
WSAS sub-scales	72	
Mild functional impairment		31 (43%)
Moderately severe functional impairment		18 (25%)
Severe functional impairment		23 (32%)
KDQOL-36 SF1.3 SF-12 Physical Health Composite	71	40 (12), 38 (31, 52)
SF-12 Mental Health Composite	71	43 (11), 42 (36, 54)
Burden of kidney disease (k = 4)	72	66 (29), 75 (44, 94)
Symptoms/problems (k = 12)	72	75 (20), 78 (64, 89)
Effects of kidney disease (k = 8)	71	78 (22), 82 (70, 94)

^a^Mean (SD), Median (IQR)

### Prior cardiovascular events and risk factors

A history of major adverse cardiovascular events affected the minority of participants (See Table 4); prior stroke and myocardial infarction (MI) were present in 1 and 3% of the cohort, respectively. There was history of heart failure (HF), and peripheral vascular disease was recorded for only 3% of participants. At baseline, 68% had hypertension, 30% had diabetes, 35% had hyperlipidaemia and 75% had never smoked.

## Discussion

Adequately powered studies that have investigated functional improvement in non-anaemic iron deficient CKD patients treated with IV iron are warranted. To date, there is a dearth of data, and a shortfall of knowledge, to address important patient reported symptoms of fatigue and exercise capacity in patients with CKD, in contrast to abundant and increasing data in the heart failure population [14].

Explorative studies that examine muscle function and structure, including mitochondrial function, may provide mechanistic insights into the role of iron therapy as a potential intervention to target the disease-related derangements in the oxygen transport chain. Investigating the further effects of an exercise training programme will provide insight into potential complementary interventions to promote physical function and capacity.

This randomised double blinded placebo-controlled trial may inform clinical practice in the management of non-anemic CKD patients with iron deficiency, as per current guidelines, to improve exercise capacity, and potentially improve quality of life and symptoms of fatigue.

The baseline demographic data of the iron and Muscle trial cohort revealed a more representative sample from all ethnic origins, when compared with previous studies [37]. There is a known under-representation of ethnic minorities in clinical trials [38–40], and it is reassuring that our sample is representative of the clinical population. All clinical values were within normal parameters and were similar to values in previous studies [37].

The results of the Iron and Muscle trial will address a significant knowledge gap in the promotion and prescription of iron therapy for patients with CKD, and the potential role of exercise training as a complementary therapy to further reduce exertional fatigue.

## Data Availability

Findings from the study will be disseminated at national and international conferences. All baseline data generated or analysed during this study are included in this published article.

## Abbreviations

IMP: Investigational medicinal product
LFT: Liver function tests
U&E: Urea and electrolytes
CRP: C-reactive protein
6MWD: 6min walk test distance
VO_2_peak: Volume of oxygen consumption
eGFR: Estimated glomerular filtration rate
Hb: Haemoglobin
TSAT: Transferrin saturation
WSAS: Work and social adjustment scale
KDQOL36: SF1.3 Kidney disease quality of life 36 questionnaire
STS60: Sit to stand 60
